# Internationalization of universities: the need to navigate in foreign waters

**DOI:** 10.1590/S1516-31802013000100001

**Published:** 2013-02-01

**Authors:** Alessandro Wasum Mariani, Paulo Manuel Pêgo-Fernandes, Marcos Naoyuki Samano

**Affiliations:** I MD. Thoracic Surgeon, Instituto do Coração (InCor), Hospital das Clínicas (HC), Faculdade de Medicina da Universidade de São Paulo (FMUSP), São Paulo, Brazil.; II MD, PhD. Associate Professor, Discipline of Thoracic Surgery, Instituto do Coração (InCor), Hospital das Clínicas (HC), Faculdade de Medicina da Universidade de São Paulo (FMUSP), São Paulo, Brazil.; III MD. Attending Physician in the Lung Transplantation Group, Instituto do Coração (InCor), Hospital das Clínicas (HC), Faculdade de Medicina da Universidade de São Paulo (FMUSP), and Collaborating Professor, Faculdade de Medicina da Universidade de São Paulo (USP), São Paulo, Brazil.

Universities around the world are increasingly interested in propagating and receiving knowledge not only to and from outside their walls but also to and from outside of their national borders. This phenomenon of interchange between institutions in different countries has become known as internationalization and many people regard this as having immense importance for full development of teaching.

Building links among researchers and knowledge-shapers from different parts of the world is extremely beneficial, since this not only makes it possible to exchange experiences, thus serving as mutual assistance with savings in time and resources in developing projects that are already in progress, but also provides impetus towards promoting and developing ideas that are completely new, emanating from these encounters.

In the view of many people, internationalization of universities is, in a certain manner, a return to their origins. As Krawczyk puts it: “It can be seen that originally, during the medieval period, universities had a strong international nature and that as a consequence of construction of modern nation-states, they underwent a process of nationalization.”^1^ Nonetheless, the objectives and the instruments through which this process has resumed its importance are noticeably different.

The motivation for the present phase of internationalization is closely related to the concept created by Slaughter and Leslie that is known as “academic capitalism”, in which researchers and university administrators are induced to participate in increasingly competitive environments, for fundraising of any nature.^1^

Information technology has played the role of an instrument for reducing the distances between institutions and the people involved, through the use of simple and cheap tools such as exchanges of e-mails, or tools of greater complexity and high cost, such as holding teleconferences between different participating locations. Nonetheless, real contact with promotion of meetings, or visits by a certain researcher to another center, is still fundamental to this process.

This is a scenario within which Brazilian universities still only have modest activity. However, a variety of initiatives have been indicating a clear change of attitude, with the aim of putting these institutions into an international context.

One good example of this is the recent forum promoted by the National Association of Directors of Federal Higher Education Institutions (Associação Nacional dos Dirigentes das Instituições Federais de Ensino Superior, ANDIFES), which dealt specifically with internationalization. The nuances of this process were discussed at this forum, and some proposals for internationalization of the federal universities were presented. The participants in this debate included not only representatives of these universities, but also representatives of the Ministry of Foreign Relations, Ministry of Education, Ministry of Science, Technology and Innovation and the National Council for Scientific and Technological Development (Conselho Nacional de Desenvolvimento Científico e Tecnológico, CNPq), among others.

It is also worth citing an important initiative from the federal government: the creation of the Science Without Borders program, which envisages awarding 100,000 bursaries for interchanges of undergraduate and postgraduate students, along with funding to attract researchers from abroad who wish to settle in Brazil or establish partnerships with Brazilian researchers.

It is not just the federal universities that are working towards internationalization. At the University of São Paulo (Universidade de São Paulo, USP), the Office of the Executive Vice-Rector for International Relations (Vice-Reitoria Executiva de Relações Internacionais, VRERI) was instituted with the aims of intensifying, stimulating and supporting the actions of internationalization. This entity within the administration acts among the various deans’ offices and teaching and research units, to provide assistance in this regard. USP maintains agreements signed with more than 50 institutions around the world. However, in the past year alone, certain actions deserve to be highlighted, such as participation in the federal Science Without Borders program, the program of interchange bursaries for undergraduate students and the program for students from foreign universities, which will award 50 bursaries to Latin American students. With the aim of achieving partnerships in cutting-edge research, the following initiatives deserve to be highlighted: creation of the University Global Partnership Network (UGPN), a special collaborative program between USP, University of Surrey in the United Kingdom and North Carolina State University (NCS) in the United States; and new agreements with three important foreign universities: University of Michigan (United States), University of Oxford (United Kingdom) and University of Toronto (Canada).

While internationalization in Brazil is still an embryonic issue, for the major universities worldwide it is a vital strategic issue that is heavily explored. The World University Rankings are produced by the magazine Times Higher Education with the aim of listing the best universities worldwide. Among the criteria used, the degree of internationalization of the institutions assessed is analyzed and scored positively. For example, around 19% of the students at Harvard University (ranked in second place in 2011-2012) are foreign; and around 21% of the students at Stanford University (ranked in third place in 2011-2012) are foreign.^2^ This is one of the factors put forward for explaining the lower performance of Brazilian universities in this ranking.

One important point regarding internationalization that may have an impact in Brazil is the fact that universities in other countries are prospecting for human material inside Brazil: Yale, Princeton and Harvard are some of the examples. All the indications are that this interest is reciprocal: increasing numbers of Brazilian students have been seeking opportunities in renowned institutions outside of this country. The University of Columbia, which has some offices representing it in Brazil for exchanges, was interviewing around 20 candidates per year up to 2007. This number leapt to 60 in 2008 and reached 100 in 2011, of whom 15 were selected. This meant that 10% of the foreign freshmen at the University of Columbia in 2012 were Brazilian.

In 2011, the rector of Harvard University came to Brazil to attempt to enter into partnerships, especially in strategic areas such as environmental management. In 2012, a delegation from the American Association of State Colleges and Universities, an entity that represents more than 400 higher education institutions in the United States, was in Brazil to try to expand its partnerships with Brazilian institutions.

As stated by José Marques dos Santos, of the University of Porto, internationalization “is not an end in itself, but an instrument that today is indispensable for fulfilling the strategic objectives that emanate from each university’s mission”.

However, in this process of internationalization, Brazil will gain little benefit if it does not take advantage of this process to attempt to make its own research centers equal to those of the greatest centers worldwide, thereby generating innovation and wealth for the country. In this regard, one good example that might be followed is that of South Korea, which in less than half a century rose from poverty to the category of economic power. This was only possible through an initial process of internationalization, in which many scientists went to the United States and Europe and subsequently returned, bringing knowledge and the will to innovate, back with them in their baggage.

Internationalization is knocking on the door of Brazilian universities. This opportunity needs to be grasped and the way to successfully go through this process needs to be determined. Drawing up strategies and applying them over as short a period as possible will be fundamental for the country’s progress.

## References

[1] Krawczyk NR (2008). As Políticas de Internacionalização das Universidades no Brasil: o caso da regionalização no Mercosul [The Policies of Internationalization of the Universities in Brazil: the Case of the Regionalization of the Mercosur]. Jornal de Políticas Educacionais.

[2] The World University Rankings World University Rankings 2011-2012.

